# The impact of optimal dating on the assessment of fetal growth

**DOI:** 10.1186/s12884-021-03640-9

**Published:** 2021-02-27

**Authors:** N. Fries, F. Dhombres, M. Massoud, J. J. Stirnemann, R. Bessis, G. Haddad, L. J. Salomon

**Affiliations:** 1 Collége Français d’Echographie Foetale, CFEF, 34820 Teyran, France; 2 Assistance Publique-Hôpitaux de Paris, Hôpital Trousseau, Sorbonne Université, Paris, France; 3 Hôpital Femme Mère Enfant et Université Claude Bernard Lyon 1, 69500 Bron, France; 4 EA FETUS, 7328, Université Paris-Descartes, Paris, France; 5 Assistance Publique-Hôpitaux de Paris, Hôpital Necker-Enfants Malades, Université de Paris, 149, Rue de Sèvres, Cedex 15, 75743 Paris, France

**Keywords:** Fetal, Growth, CRL, EFW, Integrowth, Dating

## Abstract

**Background:**

The impact of using the Intergrowth (IG) dating formulae in comparison to the commonly used Robinson dating on the evaluation of biometrics and estimated fetal weight (EFW) has not been evaluated.

**Methods:**

Nationwide cross-sectional study of routine fetal ultrasound biometry in low-risk pregnant women whose gestational age (GA) had been previously assessed by a first trimester CRL measurement. We compared the CRL-based GA according to the Robinson formula and the IG formula. We evaluated the fetal biometric measurements as well as the EFW taken later in pregnancy depending on the dating formula used. Mean and standard deviation of the Z scores as well as the number and percentage of cases classified as <3rd, < 10th, >90th and > 97th percentile were compared.

**Results:**

Three thousand five hundred twenty-two low-risk women with scans carried out after 18 weeks were included. There were differences of zero, one and 2 days in 642 (18.2%), 2700 (76.7%) and 180 (5%) when GA was estimated based on the Robinson or the IG formula, respectively. The biometry Z scores assessed later in pregnancy were all statistically significantly lower when the Intergrowth-based dating formula was used (*p* < 10^− 4^). Likewise, the number and percentage of foetuses classified as <3rd, < 10th, >90th and > 97th percentile demonstrated significant differences. As an example, the proportion of SGA foetuses varied from 3.46 to 4.57% (*p* = 0.02) and that of LGA foetuses from 17.86 to 13.4% (*p* < 10^− 4^).

**Conclusion:**

The dating formula used has a quite significant impact on the subsequent evaluation of biometry and EFW. We suggest that the combined and homogeneous use of a recent dating standard, together with prescriptive growth standards established on the same low-risk pregnancies, allows an optimal assessment of fetal growth.

## Strengths and limitations

- Analysis of a large national cohort of ultrasound data from 18 weeks of gestation onwards
- Low-risk patient who had previously undergone a crown rump length (CRL) ultrasound measurement in the first trimester.
- Evaluation of the impact of the dating reference for later estimation of fetal growth in routine practice
- No similar study available in the literature
- No correlation with perinatal outcome

## Introduction

The early detection of intrauterine growth retardation remains an important objective of antenatal ultrasound monitoring [1–4]. Yet this screening is unsatisfactory [5, 6]. Besides the problem of consensus on the tools or definitions to be used, the inappropriate use of growth curves could also be an obstacle to improving our screening [7, 8]. Inappropriate use of curves may be related to the choice of charts with several methodological limitations [7, 9], expected-value bias [10] or to the failure to respect criteria that are essential to the use of such charts: appropriate dating [11, 12] and standardized anatomical sections with well-defined biometric landmarks [8, 13, 14].

As part of the INTERGROWTH-21st (IG) Project, the International Foetal Growth Standards were published in 2014 [15]. These standards were elaborated within the framework of a prospective, multi-ethnic, international and population-based research project that initially selected urban areas on all five continents. Most of the people living in the selected areas were healthy, well-nourished and educated, with limited environmental constraints on growth. In a second sampling stage, pregnant women were recruited from these study sites, whose health, nutrition and care needs were met, ensuring that fetal growth was as optimal as possible. This procedure follows the same conceptual, methodological and analytical concepts used in the creation of the WHO Child Growth Standards, which makes it possible to monitor growth and development using high-quality tools during the first 1000 critical days and up to the age of 5 years. In comparison to previous locally produced references, international standards have the potential to enhance the detection of growth disorders and, consequently, perinatal outcomes, by the standardisation of the diagnostic approach to IUGR and macrosomia. In addition, the study provides a variety of new tools that allow unified monitoring, such as GA estimation, fetal growth, Doppler, height and neonatal development at 2 years of age.

These standards were established using an last menstruation period (LMP) gestational age (GA) estimation [15]. Enrollment was prospective and consecutive from 9 + 0 to 13 + 6 weeks gestation, according to LMP’s estimates, provided that: (1) the LMP was certain; (2) the agreement between LMP and CRL on the date was ≤7 days; (3) the women were having a regular menstrual cycle of 24 to 32 days; and (4) they were not using hormonal contraception or breastfeeding in the previous 2 months. However, in a general population of women such as we care for every day, there is a broad consensus to date pregnancies on the basis of the CRL [1, 12, 16, 17], which makes it possible to avoid memory errors [18, 19], which are very frequent, but also the uncertainty associated with irregular cycles. Such CRL-based assessment of GA is recommended by most professional societies [1, 12, 16, 17].

On the basis of the IG cohort, CRL-based standards for the estimation of GA were also established [20]. This standard is very close to the reference developed by Robinson more than 30 years ago and which is still widely used [21]. However, logic would dictate that dating based on the formula developed on the IG cohort should be used when later foetal biometric measurements are to be evaluated using these same cohort-based standards.

We sought to assess the impact of using IG dating in comparison to Robinson dating on the evaluation of biometrics and EFW, in a large nationwide cohort of women.

## Material and methods

This study was based on data gathered during the CFEF Flash biometric study and already reported elsewhere [22]. Briefly, Flash studies are pragmatic, short and very focused studies, conducted without modifying routine clinical practice and at no extra cost. They have both a scientific and an educational purpose and are conducted in France across the countrywide network of sonographers who are members of the French College of Foetal Ultrasound (College Français d’Echographie Foetale (CFEF)). For this study, we had invited sonographers first to take an online training course (www.cfef.org) that reviewed the aims of the study, the inclusion criteria, the methodology for taking the measurements and the biometric quality control criteria. Only sonographers who had completed the course and passed the final test were eligible to participate. All participating sonographers had, after oral explanations, to obtain the women’s oral informed consent to the fully anonymized use of fetal biometric data collected during routine examinations. Pregnant women, after oral informed consent, contributed with a single measure and were included prospectively and consecutively over a fixed study period of 6 weeks. Those included had a singleton pregnancy without congenital malformations and with a documented dating based on crown–rump length measurement in the first trimester, as recommended by the French College of Obstetrics and Gynaecology and performed according to commonly agreed quality criteria [17, 20, 23]. The data were entered anonymously by the participating sonographers. No co-authors were therefore involved in the anonymization process. These measurements, collected prospectively, constituted our primary database. Within this dataset, a subsample of low-risk women was selected as those who met, as closely as possible, the strict inclusion criteria of the Foetal Growth Longitudinal Study (FGLS) of the INTERGROWTH-21st Project, as described previously. More details about methods for recruitment, collected information and foetal measurements techniques can be found elsewhere [22] . This study was carried out as part of routine care and did not change the patient’s management. In accordance with French laws in force at the time the biometric data of the initial study were collected, such a study did not require an IRB. For the purpose of the current study, only patients with biometric measurements from 18 weeks onwards were used.

We evaluated the impact of the GA-estimation method as follows: we analysed the distribution of CRL measurements in the first trimester and compared the estimated gestational age based on the Robinson formula and the IG formula. The number and % of cases strictly concordant for GA or within one, two or more days difference were evaluated. We then evaluated the foetal biometric measurements as well as the EFW calculated by the Hadlock formula (both according to the IG standard [15, 24]), taken later in pregnancy depending on the dating formula used. The mean and standard deviation of the Z scores as well as the number of cases classified as <3rd, < 10th, >90th and > 97th percentile were calculated and compared by means of paired-t test and McNemar’s test, respectively.

We calculated that demonstrating a difference of 0.1SD in the mean Z score of measurements would require about 1500 observations and that a 2% change in the proportion of foetuses considered smaller than the 10th percentile or larger than the 90th percentile would require about 3500 observations, both with alpha and beta set at 5 and 20%, respectively.

Statistical analyses were performed using Stata 9.2 for Windows (StataCorp LP, College Station, TX, USA), Statistica (StatSoft, Inc., 2001).

## Results

**Fig. 1:**
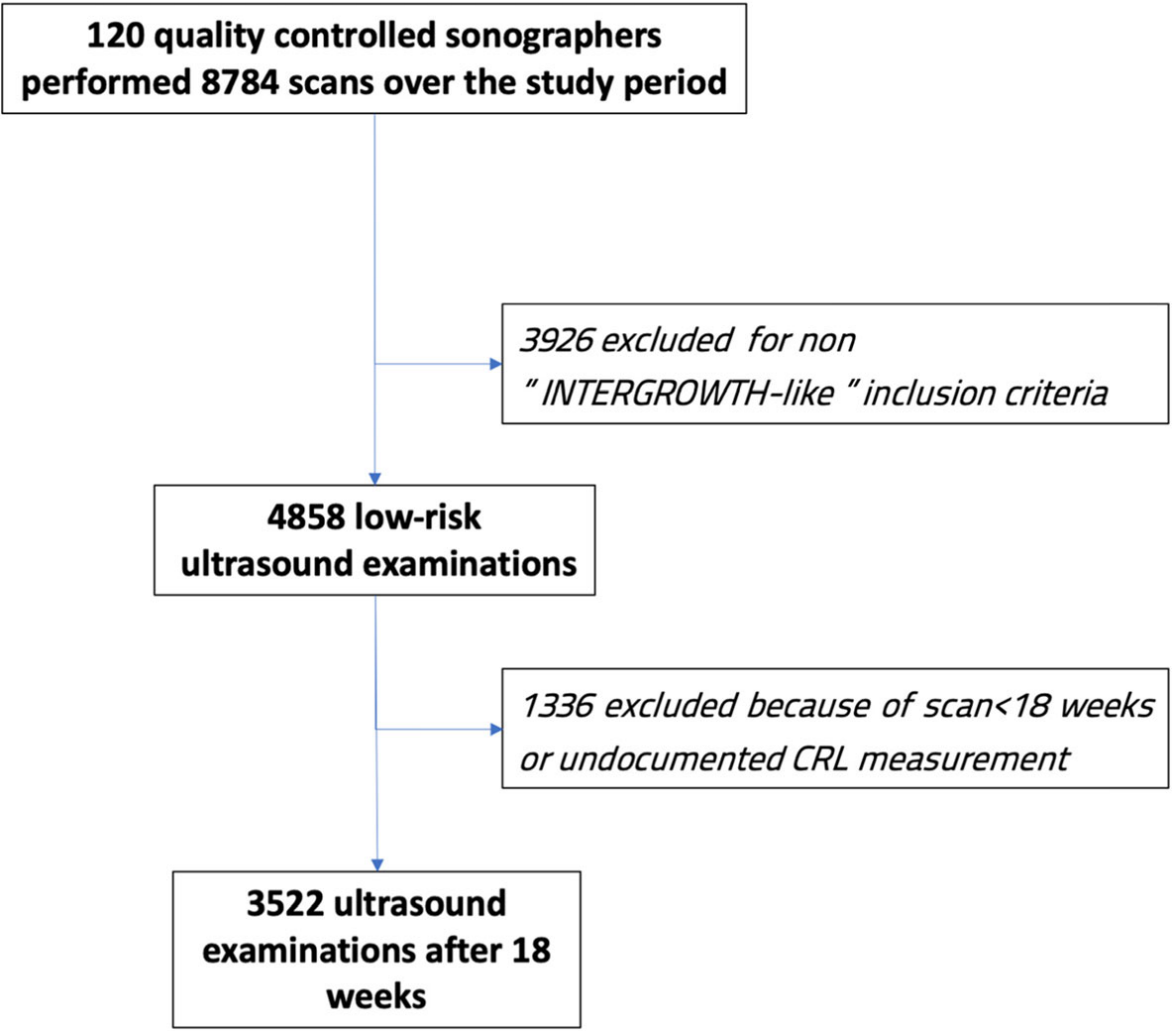
Flow chart

As previously communicated, 160 ultrasound practitioners agreed to collaborate, 120 of whom met the requirements for inclusion in the study. During the period of the study, they completed a total of 8784 scans. We then selected 4858 (55.3%) independent ultrasound scans in women and fetuses at low risk during gestation, i.e. a population of “INTERGROWTH-21th FGLS” type. After excluding examinations before 18 weeks or with undocumented CRL measurement, there were 3522 cases remaining (Fig. 1: flow chart).

As expected from the examination of the two references [20, 21], there was no difference in estimated gestational age for CRL of less than 55 mm, a difference of 1 day for CRL of 55 to 75 mm, and a difference of 2 days for CRL greater than 75 mm.

**Fig. 2:**
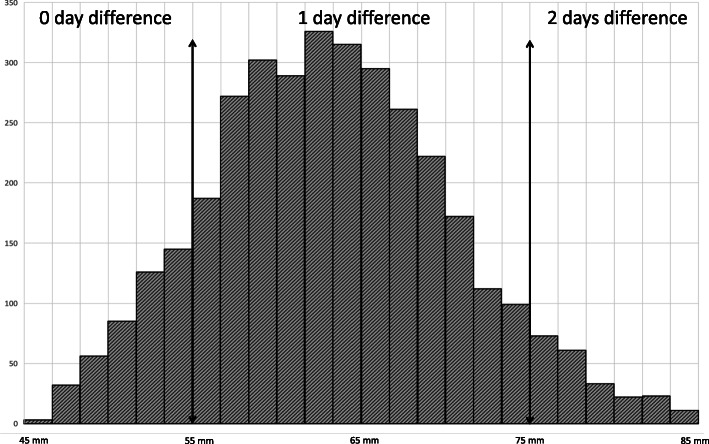
Observed distribution of CRL measurements in the first trimester and associated GA differences according to the two references [20, 21]

The observed distribution of CRL in the first trimester in our general population is shown on Fig. 2.

Expectedly, examinations were preferably performed around the centre of the recommended examination period for CRLs in between 45 and 84 mm. This led to differences of zero, one and 2 days in 642 (18.2%), 2700 (76.7%) and 180 (5%) when the gestational age was estimated based on the Robinson or the IG formula, respectively. There was no case with a GA estimation difference of more than 2 days. Overall, the average dating difference was 0.87+/− 0.47 days. Where there was a difference, it was always in the same direction that the IG standard estimated the pregnancy one or 2 days more advanced than the Robinson’s reference.

The biometry Z scores assessed later in pregnancy were all statistically significantly lower when the Intergrowth-based dating formula was used (Table 1). Likewise, the number and percentage of foetuses classified as <3rd, < 10th, >90th and > 97th percentile were calculated and compared (Table 2) and demonstrated significant differences for all but one comparison (FL below the third centile, *p* = 0.08). Overall, the results observed by applying IG-based GA assessment indicated means and SDs closer to the expected values of 0 and 1, respectively. Likewise, the % of fetuses considered small or large were also closer to the expected values.

**Table 1:** Head circumference (HC), Abdominal circumference (AC), Femur length (FL) and Estimated Fetal Weight (EFW) values expressed as mean and SD of Z-scores according to the two different GA estimation [20, 21] and paired t-test comparison

	Robinson-based GA assessment	Intergrowth-based GA assessment	Within-subject differences^a^: Mean (SD)	***p value***
**Head circumference (HC)** **(*n*** **= 3522)**	−0.0251 (1.057)	− 0.1484 (1.056)	−0.1251 (0.075)	***< 10***^***− 4***^
**Abdominal circumference (AC)** **(*n*** **= 3522)**	0.4808 (0.990)	0.3675 (0.979)	−0.1150 (0.065)	***< 10***^***−*** *4*^
**Femur length (FL)** **(*n*** **= 3522)**	0.5236 (1.021)	0.3989 (1.021)	−0.1267 (0.072)	***< 10***^***−4***^
**Estimated foetal weight (EFW)** **(*n*** **= 3522)**	0.4424 (1.008)	0.2898 (1.005)	−0.1548 (0.090)	***< 10***^***−4***^

^a^ *Mean (SD) for each biometric measurement were calculated by subtracting the value obtained using the Robinson dating formula from the value obtained using the Intergrowth dating formula within-person and then taking the mean and SD of these differences*

**Table 2:** Number and percentage of Head circumference (HC), Abdominal circumference (AC), Femur length (FL) and Estimated Fetal Weight (EFW) classified as <3rd, < 10th, >90th and > 97th percentile according to the two difference GA estimation [20, 21]. The dataset is defined by the four cell totals a, b, c, and d for the dichotomous variables. The values in a (condition fulfilled) and d (condition not fulfilled) are the concordant cells; those in b and c are the discordant cells^a^. *p* value given from the McNemar’s test

		***Robinson-based GA assessment*** ***n (%)***	***Intergrowth-based GA assessment*** ***n (%)***	***a***	***b***	***c***	***d***	***p value***
**Head circumference (HC)** **(*n*** **= 3522)**	<3rd centile	112 (3.18)	135 (3.83)	112	23	0	3387	**< 10**^**− 4**^
	< 10th centile	341 (9.68)	413 (11.73)	341	72	0	3109	**< 10**^**−4**^
	>90th centile	334 (9.48)	274 (7.78)	274	0	60	3188	**< 10**^**−4**^
	>97th centile	113 (3.21)	94 (2.67)	94	0	19	1409	**< 10**^**−4**^
**Abdominal circumference (AC)** **(*n*** **= 3522)**	<3rd centile	34 (0.97)	40 (1.14)	34	6	0	3482	**0.01**
	< 10th centile	130 (3.69)	152 (4.32)	130	22	0	3370	**< 10**^**−4**^
	>90th centile	731 (20.7)	595 (16.8)	595	0	136	2791	**< 10**^**−4**^
	>97th centile	248 (7.04)	191 (5.42)	191	0	57	3274	**< 10**^**−4**^
**Femur length (FL)** **(*n*** **= 3522)**	<3rd centile	32 (0.91)	34 (0.99)	32	3	0	3487	0.08
	< 10th centile	91 (2.58)	117 (3.32)	91	26	0	3405	**< 10**^**−4**^
	>90th centile	736 (20.90)	621 (17.63)	621	0	115	2786	**< 10**^**−4**^
	>97th centile	285 (8.09)	236 (6.70)	236	0	49	3237	**< 10**^**−4**^
**Estimated foetal weight (EFW)** **(*n*** **= 3522)**	<3rd centile	55 (1.56)	64 (1.82)	55	9	0	3458	**0.002**
	< 10th centile	122 (3.46)	161 (4.57)	122	39	0	3361	**< 10**^**−4**^
	>90th centile	629 (17.86)	472 (13.40)	472	0	157	2893	**<10**^**−4**^
	>97th centile	191 (5.42)	124 (3.52)	124	0	67	3331	**<10**^**−4**^

*GA* Gestational age*, SD* Standard deviation*, EFW* Estimated foetal weight*.*

^a^ *b = condition fulfilled using Intergrowth-based GA assessment but not Robinson-based GA assessment, and c = condition fulfilled using Robinson-based GA assessment but not Intergrowth-based GA assessment*

## Discussion

Our study shows that the dating formula used has a quite significant impact on the subsequent evaluation of biometry and estimation of foetal weight. Although the two formulas used here seem theoretically very similar at first glance, our data suggests that when used in real life, they can make the number of foetuses considered too small or too large vary significantly.

Optimal assessment of foetal growth and screening for growth retardation are complex and difficult processes. They must be based on perfectly constructed and standardized tools. It is essential that growth standards, showing how a foetus should grow, are used. These prescriptive standards are now widely recommended over descriptive local references [1, 25–28]. When developing such standards, it is also desirable that reliable information on LMP rather than CRL measurement alone, are used as the basis for GA assessment: this is particularly true as ultrasound dating has not demonstrated more accurate than a reliable LMP confirmed by CRL and because CRL variations may reflect early differences in foetal growth [29]. Nonetheless, outside of this context of prescriptive growth standard development, at the individual level, there is a broad consensus around the world to accurately assess the age of pregnancy and to base this assessment on the measurement of CRL in the first trimester [11, 12, 30, 31]. Precise CRL measurement and CRL-based dating is of utmost importance to interpret first-trimester screening for chromosomal abnormalities, to reduce the number of pregnancies classified as preterm and to reduce the number of unnecessary inductions of labour or post-term delivery [18, 23, 32–36]. There is variation in practice and no consensus exists on which formula is the most appropriate for pregnancy dating. The Robinson’s reference, although being developed in 1975 from only 334 foetuses scanned transabdominally between 6 and 12 weeks of gestation in the suburbs of Glasgow has become an almost universally accepted reference that appears robust in daily practice [21]. In a comprehensive review of existing CRL curves, this reference was also selected among the four with the least risk of bias [32]. However, the newly issued first trimester IG standard was based on a very broad international population of women from eight geographically distinct areas of the world, with very little constraints on fetal growth at the population and individual level, in order to build standards for CRL and the corresponding GA estimate in the first trimester of pregnancy. It was a population-based, prospective study that included only single and naturally conceived pregnancies with known LMP in women with a 24–32 day regular menstrual cycle and who were not using hormonal contraception or breastfeeding in the previous 2 months. Although the Robinson formula does not differ by more than 2 days from the new IG standard, our study demonstrates that switching from one reference to another may have a significant impact. In our test-population, using the new IG standard instead of the Robinson equation resulted in means and standard deviations of Z-scores closer to the respective anticipated values of 0 and 1. Similarly, the percentages of foetuses considered as small or large for GA were closer to the expected values when using the new IG standard for GA assessment. This suggests that the use of a dating standard that matches the growth standards used subsequently allows a more accurate assessment of foetal growth. Interestingly, some recent studies have suggested that using the new IG standard could tend to classify too many foetuses as large and too few as small [37–39]. This is undoubtedly related to the fact that populations tend to have increasingly larger foetuses [40, 41], which is particularly noticeable when these foetuses are no longer compared to descriptive but prescriptive curves. Our report shows that this tendency decreases significantly once pregnancy dating is carried out with the new IG CRL standard. Indeed, in addition to the fact that these studies were not based on the estimation of foetal weight obtained from the Hadlock formulae [42] and the corresponding IG standard [24], these studies also did not use a determination of GA based on the Intergrowth CRL standard. The possibility to evaluate GA based on a recent, quality-checked CRL reference and then to assess biometry using a prescriptive growth reference, established on the very same population whose gestational age had been established for the development of the standards on the most physiological markers (LMP), is a unique combination and allows a homogeneous and consistent assessment of foetal growth.

It has been previously emphasized that different methods of assigning gestational age affect the assessment of foetal measurements and of birth weight for gestational age [43, 44]. On the other hand, we are not aware of any study that has specifically evaluated the impact of using either of the CRL references, and it is frequently considered that GA assessment based on first trimester biometry is sufficient for the subsequent assessment of growth [30]. Our study confirms that the consistency of CRL measurement together with the choice of the reference equation cant induce heterogeneity in gestational age estimation and affect the accuracy of subsequent foetal biometry [45]. Previous CRL references, such as the Robinson one, were often performed on small monocentric populations, with unknown pregnancy outcomes, by a single observer, without quality control, and on ultrasound devices whose performance has greatly evolved. On the opposite, the IG standards were developed from a multi-ethnic populations worldwide, whose health, nutrition and care needs were largely met, under strict quality control criteria and with recent ultrasound machines.

### Strengths and limitations

A strength of this analysis is that it involves a large panel of sonographer, undergoing quality control and in real life situation. It is pragmatic and directly describes the effect of applying the new IG CRL standard. However, some limitations in this study should be acknowledged. Sonographers who were volunteers and eventually were enrolled in this study may not fully represent the general population of sonographers. They have also performed CRL and biometric measurements in a non-blinded fashion, comparing them to existing local references that may have introduced a bias towards the expected values. Moreover, we did not assess variability across the 120 sonographers. For the same reason that all gestational age differences as assessed based on Robinson and Intergrowth were in the same direction; all biometric or EFW differences were in the same direction at all GAs. However, we did not attempt to test for possible interaction with GA. Finally, we have not collected birth weights nor perinatal outcomes that could have suggested that measurements taken with the IG standard for CRL, which are more closely aligned with expected values, are also more predictive of perinatal outcome.

## Conclusion

We believe that the combined use of a recent dating standard, together with prescriptive growth standards established on the same low-risk pregnancies and dated on LMP, allows an optimal assessment of foetal growth. Our study shows that the use of the same set of tools for dating, biometrics and EFW is important and should be favoured over the use of heterogeneous references of diverse origins. Assessment of foetal growth is difficult and screening for growth abnormalities remains poor. In order to optimize this screening, it is essential to standardize the tools used, in order to limit as much as possible the bias at each and every step. Such a homogeneous approach based on perfectly standardized and mutually calibrated tools can be undertaken and extended with the different Intergrowth standards which, once implemented as a whole, have the potential to ensure consistent assessment of the foetus and then of the new-born and the child [27, 28].

## Data Availability

The datasets used and/or analysed during the current study available from the corresponding author on reasonable request. (NF, LJS).

## References

[1] Salomon LJ, Alfirevic Z, Da Silva Costa F (2019). ISUOG Practice guidelines: ultrasound assessment of fetal biometry and growth. Ultrasound Obstet Gynecol.

[2] Lees CC, Stampalija T, Baschat A (2020). ISUOG Practice guidelines: diagnosis and management of small-for-gestational-age fetus and fetal growth restriction. Ultrasound Obstet Gynecol.

[3] ACOG Practice Bulletin No. 204 summary (2019). Fetal growth restriction. Obstet Gynecol.

[4] Martins JG, Biggio JR, Abuhamad A. Society for maternal-fetal medicine (SMFM) consult series #52: diagnosis and management of fetal growth restriction. Am J Obstet Gynecol. 2020:S0002937820305354. 10.1016/j.ajog.2020.05.010. 10.1016/j.ajog.2020.05.010 32407785

[5] Monier I, Blondel B, Ego A (2015). Poor effectiveness of antenatal detection of fetal growth restriction and consequences for obstetric management and neonatal outcomes: a French national study. BJOG.

[6] Sovio U, White IR, Dacey A (2015). Screening for fetal growth restriction with universal third trimester ultrasonography in nulliparous women in the pregnancy outcome prediction (POP) study: a prospective cohort study. Lancet.

[7] Ioannou C, Talbot K, Ohuma E, et al. Systematic review of methodology used in ultrasound studies aimed at creating charts of fetal size. BJOG. 2012. 10.1111/j.1471-0528.2012.03451.x. 10.1111/j.1471-0528.2012.03451.x 22882780

[8] Salomon LJ, Bernard JP, Duyme M (2005). The impact of choice of reference charts and equations on the assessment of fetal biometry: assessment of fetal biometry. Ultrasound Obstet Gynecol.

[9] Sarris I, Ioannou C, Dighe M (2011). Standardization of fetal ultrasound biometry measurements: improving the quality and consistency of measurements. Ultrasound Obstet Gynecol.

[10] Drukker L, Droste R, Chatelain P (2020). Expected-value bias in routine third-trimester growth scans. Ultrasound Obstet Gynecol.

[11] Vayssière C, Haumonte J-B, Chantry A (2013). Prolonged and post-term pregnancies: guidelines for clinical practice from the French College of Gynecologists and Obstetricians (CNGOF). Eur J Obstet Gynecol Reprod Biol.

[12] Salomon LJ (2011). How to date pregnancy?. J Gynecol Obstet Biol Reprod (Paris).

[13] Dudley NJ, Griffith K (1996). The importance of rigorous testing of circumference measuring callipers. Ultrasound Med Biol.

[14] Napolitano R, Donadono V, Ohuma EO (2016). Scientific basis for standardization of fetal head measurements by ultrasound: a reproducibility study. Ultrasound Obstet Gynecol.

[15] Papageorghiou AT, Ohuma EO, Altman DG (2014). International standards for fetal growth based on serial ultrasound measurements: the fetal growth longitudinal study of the INTERGROWTH-21st project. Lancet.

[16] Vayssière C, Sentilhes L, Ego A (2015). Fetal growth restriction and intra-uterine growth restriction: guidelines for clinical practice from the French College of Gynaecologists and Obstetricians. Eur J Obstet Gynecol Reprod Biol.

[17] Salomon LJ, Alfirevic Z, Seshadri S, et al. ISUOG Practice Guidelines: performance of first-trimester fetal ultrasound scan. Ultrasound Obstet Gynecol. 2013;41(1):102–13. 10.1002/uog.12342. 10.1002/uog.12342 23280739

[18] Savitz DA, Terry JW, Dole N (2002). Comparison of pregnancy dating by last menstrual period, ultrasound scanning, and their combination. Am J Obstet Gynecol.

[19] Hoffman CS, Messer LC, Mendola P (2008). Comparison of gestational age at birth based on last menstrual period and ultrasound during the first trimester. Paediatr Perinat Epidemiol.

[20] Papageorghiou AT, Kennedy SH, Salomon LJ (2014). International standards for early fetal size and pregnancy dating based on ultrasound measurement of crown-rump length in the first trimester of pregnancy. Ultrasound Obstet Gynecol.

[21] Robinson HP, Fleming JE (1975). A critical evaluation of sonar “crown-rump length” measurements. Br J Obstet Gynaecol.

[22] Stirnemann JJ, Fries N, Bessis R (2017). Implementing the INTERGROWTH-21(st) fetal growth standards in France: a “flash study” of the college Français d’Echographie Foetale (CFEF). Ultrasound Obstet Gynecol.

[23] Dhombres F, Khoshnood B, Bessis R (2014). Quality of first-trimester measurement of crown-rump length. Am J Obstet Gynecol.

[24] Stirnemann J, Salomon LJ, Papageorghiou A. The Intergrowth standards for Hadlock’s estimation of fetal weight. Ultrasound Obstet Gynecol Published Online First: 22 February 2020. doi:10.1002/uog.22000. 10.1002/uog.22000 32086966

[25] Borghi E, de Onis M, Garza C (2006). Construction of the World Health Organization child growth standards: selection of methods for attained growth curves. Stat Med.

[26] WHO. Report on the regional consultation towards the development of a strategy for optimizing fetal growth and development. Cairo: World Health Organization; 2005.

[27] Papageorghiou AT, Kennedy SH, Salomon LJ (2018). The INTERGROWTH-21 st fetal growth standards: toward the global integration of pregnancy and pediatric care. Am J Obstet Gynecol.

[28] Villar J, Papageorghiou AT, Pang R (2015). Monitoring human growth and development: a continuum from the womb to the classroom. Am J Obstet Gynecol.

[29] Salomon L, Hourrier S, Fanchin R, et al. Is first-trimester crown-rump length associated with birthweight? BJOGPublished Online First: 18 May 2011. doi:10.1111/j.1471-0528.2011.03009.x. 10.1111/j.1471-0528.2011.03009.x 21585646

[30] ISUOG Practice guidelines: performance of first-trimester fetal ultrasound scan (2013). ISUOG guidelines. Ultrasound Obstet Gynecol.

[31] Committee Opinion No 700 (2017). Methods for estimating the due date. Obstet Gynecol.

[32] Napolitano R, Dhami J, Ohuma EO (2014). Pregnancy dating by fetal crown-rump length: a systematic review of charts. BJOG.

[33] Gardosi J, Geirsson RT (1998). Routine ultrasound is the method of choice for dating pregnancy. Br J Obstet Gynaecol.

[34] Harrington D, MacKenzie I, Thompson K (2006). Does a first trimester dating scan using crown rump length measurement reduce the rate of induction of labour for prolonged pregnancy? An uncompleted randomised controlled trial of 463 women. BJOG.

[35] Caughey AB, Nicholson JM, Washington AE (2008). First- vs second-trimester ultrasound: the effect on pregnancy dating and perinatal outcomes. Am J Obstet Gynecol.

[36] Dhombres F, Roux N, Friszer S (2016). Relation between the quality of the ultrasound image acquisition and the precision of the measurement of the crown-rump length in the late first trimester: what are the consequences?. Eur J Obstet Gynecol Reprod Biol.

[37] Anderson NH, Sadler LC, McKinlay CJD (2016). INTERGROWTH-21st vs customized birthweight standards for identification of perinatal mortality and morbidity. Am J Obstet Gynecol.

[38] Liu S, Metcalfe A, León JA (2017). Evaluation of the INTERGROWTH-21st project newborn standard for use in Canada. PLoS One.

[39] Choi SKY, Gordon A, Hilder L, et al. Performance of six birthweight and estimated fetal weight standards for predicting adverse perinatal outcomes: a 10-year nationwide population-based study. Ultrasound Obstet Gynecol Published Online First: 16 2020. doi:10.1002/uog.22151.

[40] Skjaerven R, Gjessing HK, Bakketeig LS (2000). Birthweight by gestational age in Norway. Acta Obstet Gynecol Scand.

[41] Ghosh RE, Berild JD, Sterrantino AF (2018). Birth weight trends in England and Wales (1986-2012): babies are getting heavier. Arch Dis Child Fetal Neonatal Ed.

[42] Hadlock FP, Harrist RB, Sharman RS (1985). Estimation of fetal weight with the use of head, body, and femur measurements--a prospective study. Am J Obstet Gynecol.

[43] Callaghan WM, Dietz PM (2010). Differences in birth weight for gestational age distributions according to the measures used to assign gestational age. Am J Epidemiol.

[44] Lynch CD, Zhang J (2007). The research implications of the selection of a gestational age estimation method. Paediatr Perinat Epidemiol.

[45] Ioannou C, Sarris I, Hoch L (2013). Standardisation of crown-rump length measurement. BJOG.

